# Computerized tomography angiography in diagnosing an obtuse marginal branch perforation after pericardiocentesis: a case report

**DOI:** 10.3389/fcvm.2025.1535797

**Published:** 2025-03-11

**Authors:** A. Ostojic, Z. Antonic, I. Ilic

**Affiliations:** ^1^ Department of Cardiovascular Imaging, Interventional Cardiology, Institute for Cardiovascular Diseases “Dedinje”, Belgrade, Serbia; ^2^ Faculty of Medicine, University of Belgrade, Belgrade, Serbia

**Keywords:** coronary laceration, aortic dissection, pericardiocentesis, computed tomography angiography, obtuse marginal

## Abstract

**Background:**

Pericardiocentesis is both therapeutic and diagnostic invasive procedure, guided by echocardiography and/or angiography. It can be done using subcostal or apical approach. One of the major complications of pericardiocentesis is coronary artery laceration with an incidence of less than 1%. Diagnosis of such lacerations is often made by invasive coronary angiography or urgent thoracotomy. Computed tomography angiography is used to determine the extent of bleeding and hemopericardium, but its potential for detailed evaluation of bleeding site is somewhat underestimated.

**Case presentation:**

We present a rare case of distal obtuse marginal (OM) artery perforation resulting from apical pericardiocentesis that was diagnosed with CT angiography (CTA) further treated with coronary guidewire particle embolization. A 49-year-old male patient who had undergone ascending aorta and aortic arch reconstruction after an aortic dissection Type A was evaluated with echocardiography before being discharged from our hospital. A loculated pericardial effusion was identified, necessitating pericardiocentesis. The clinical course was further complicated by hemopericardium due to coronary laceration. The hemorrhage was managed with coronary guidewire segment embolization which led to immediate improvement in hemodynamic status. The patient was discharged seven days after intervention.

**Conclusion:**

Coronary artery perforation is a rare, albeit life-threatening complication of pericardiocentesis that requires urgent surgical or percutaneous intervention. CTA can provide important diagnostic information on perforation location and help in deciding whether embolization or open-heart surgery is needed to address ongoing bleeding.

## Introduction

Pericardiocentesis presents a lifesaving procedure in patients with large pericardial effusion. It is a relatively safe procedure if it is done with ultrasound or fluoroscopy guidance. The complications of the procedure are infrequent and are associated with the approach used. The rate of complications depends on the distance that needle has to traverse in order to reach pericardial space (1, 2). The most frequent complications are liver injury, pneumothorax and cardiac chamber rupture (1). Iatrogenic coronary laceration is a rare complication that arises from catheter or guidewire manipulation during pericardiocentesis.

Cardiac computerized tomography angiography (CTA) is considered useful in evaluation of blunt or penetrating chest trauma, diagnosis of hemo- or pneumopericardium and coronary artery disease (3). It can be used for precise location of pericardial effusion and intervention guidance (4).

## Case presentation

A 49-year-old male patient was admitted to our hospital with acute stabbing chest pain due to aortic dissection type A involving supraaortic branches.

He had a history of drug abuse and tuberculosis but no other known comorbidities. Upon admission, the patient was hypertensive and pale. Laboratory investigations results were within normal ranges.

The patient was promptly sent to the operating room, where urgent cardiac surgery was performed, including artificial aortic valve implantation and tubular graft implantation in the ascending aorta. The brachiocephalic trunk and common carotid artery were reconstructed using tubular grafts and reimplanted into the ascending aorta tubular graft.

On the first postoperative day, the patient was surgically revised due to increased thoracic drainage and hemodynamic instability, but no bleeding site was found. Hemodynamic stability was achieved postoperatively with inotropic and vasopressor therapy as well as blood transfusion.

The patient had prolonged postoperative course with prolonged mechanical ventilation due to acute renal failure that required renal replacement therapy and ventilator associated pneumonia (VAP) that ended in tracheostomy.

One month postoperative, the patient developed sepsis with hypotension, necessitating reinitiation of vasopressor therapy. Sepsis led to further deterioration of renal function. Circular pericardial effusion, with a thickness of 20 mm, was seen on echocardiogram, but biventricular systolic function remained normal. The procalcitonin level was 110 µg/L. Colistin and Tigecycline, along with antifungal medication, were introduced into the therapy.

Two weeks later, the patient's condition had improved and he was weaned from mechanical ventilation.

Repeat echocardiography revealed a slightly decreased left ventricular ejection fraction (LVEF) of 50%. It also showed loculated pericardial effusion, with a thickness of 17 mm behind the lateral and 25 mm around the inferior and posterior wall of the left ventricle (LV). There were no significant effusion around the right heart. There were no signs of cardiac tamponade, with significant variations of pulse wave doppler signal variations with respirations.

Considering the afore mentioned echocardiogram and newly recognized pericardial effusion CT was done.

Thoracic CT, performed before and after contrast administration, revealed a circumferential collection around the ascending aorta graft, with low density (less than 10 HU) and loculated pericardial effusion near the lateral wall of the left ventricle (LV). The effusion was noted to extend cranially to the main stem of the pulmonary artery, with the maximum thickness of 38 mm.

Pericardial effusion showed no increase in density after contrast application and expanded toward lung parenchyma causing subsegmental atelectasis.

Considering the large pericardial effusion (>20 mm) and the possibility of overt cardiac tamponade, it was decided to drain the pericardial fluid.

Although CT guided pericardicentesis could be an option, patient underwent several CT scans recently with total summed effective radiation dose of 104.5 mSv and knowing that this procedure was not part of the hospital's routine practice, we opted for cath lab procedure aided with echocardiography (5).

Echocardiography and fluoroscopy guided pericardiocentesis via an apical approach was done, resulting in the removal of 300 ml of serosanguineous fluid. A 6 French pericardial catheter was placed. Post-procedural echocardiography showed almost complete resolution of the effusion, with a remaining 5 mm thickness of pericardial fluid near the lateral LV wall.

Ten hours after the procedure, the patient became hypotensive, with a blood pressure of 80/60 mmHg. A bedside transthoracic echocardiogram suggested a large pericardial effusion with a pericardial thrombus of 50 mm thickness along the lateral wall. Laboratory tests revealed a drop of 3 g/dl in hemoglobin level. The examination showed muffled heart sounds with tachycardia.

Urgent ECG triggered CTA was done in native phase and two postcontrast phases—arterial and portovenous. CT examination revealed a circumferential heterogeneous pericardial effusion with a density of 60–80 HU, consistent with hemopericardium and formation of intrapericardial hematoma, with the thickest part along the posterolateral segments of the LV wall measuring 52 mm. In the early arterial phase, linear contrast extravasation in the apicolateral myocardium segments [irrigated by the Left Circumflex artery (LCx)] was visualized, suggesting possible Type IIIA perforation (Ellis classification) of the obtuse marginal branch of LCx.

**Figure 1:**
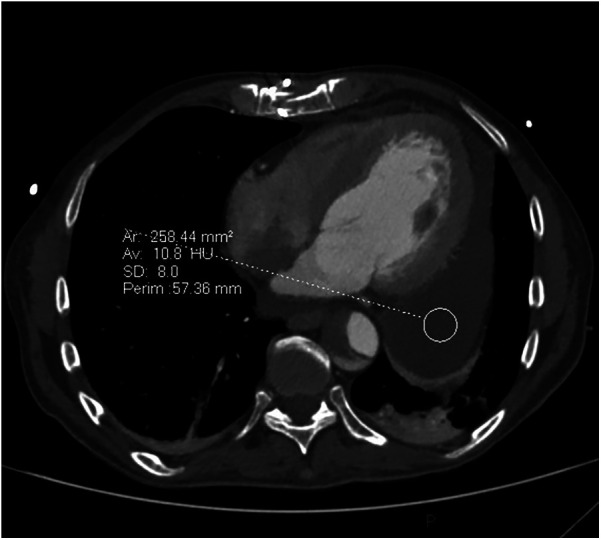
Cross section cardiac CT image before pericardocentesis demonstrating circumferential low attenuating pericardial effusion, thickest behind lateral wall of the left ventricle.

**Figure 2:**
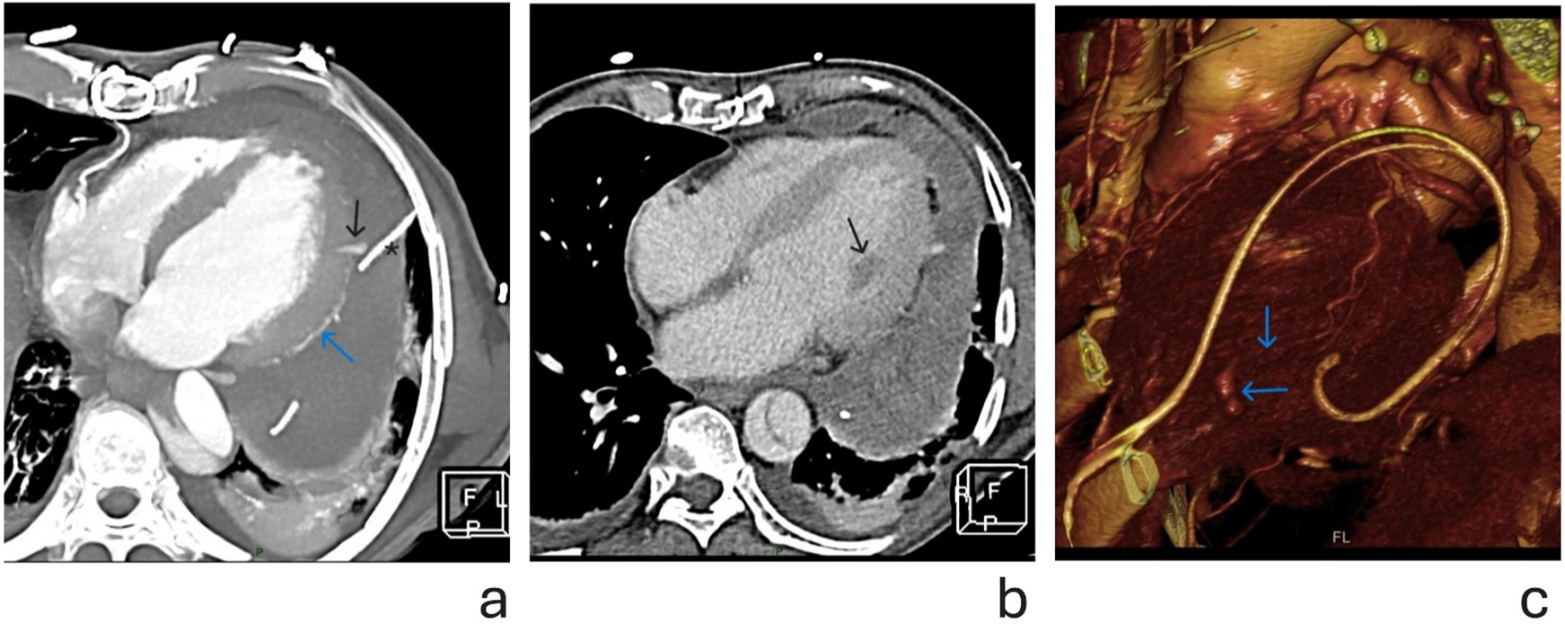
**(a)** Cross sectional thickened maximal intensity projections (MIP) image of cardiac CT after pericardiocentesis, in early arterial phase. Blue arrow shows the course of OM branch. Black arrow points to the contrast extravasation, at distal part of the OM branch. Note the proximity of the pericardial catheter (marked with *) to the site of hemorrhage. **(b)** Axial cross section CT image showing heterogeneous pericardial effusion in portovenous phase. Black arrow points to hypodense (ischemic) anterolateral papillary muscle, irrigated by OM branch, further suggesting its lesion. **(c)** Volume rendering (VR) reconstruction showing—blue arrows pointing to distal OM and site of contrast extravasation.

Additionally, the delayed post-contrast phase (portovenous phase) showed decreased myocardial opacification of the anterolateral papillary muscle, which is irrigated by the first obtuse marginal branch (OM1). (Figures 1, 2).

**Figure 3:**
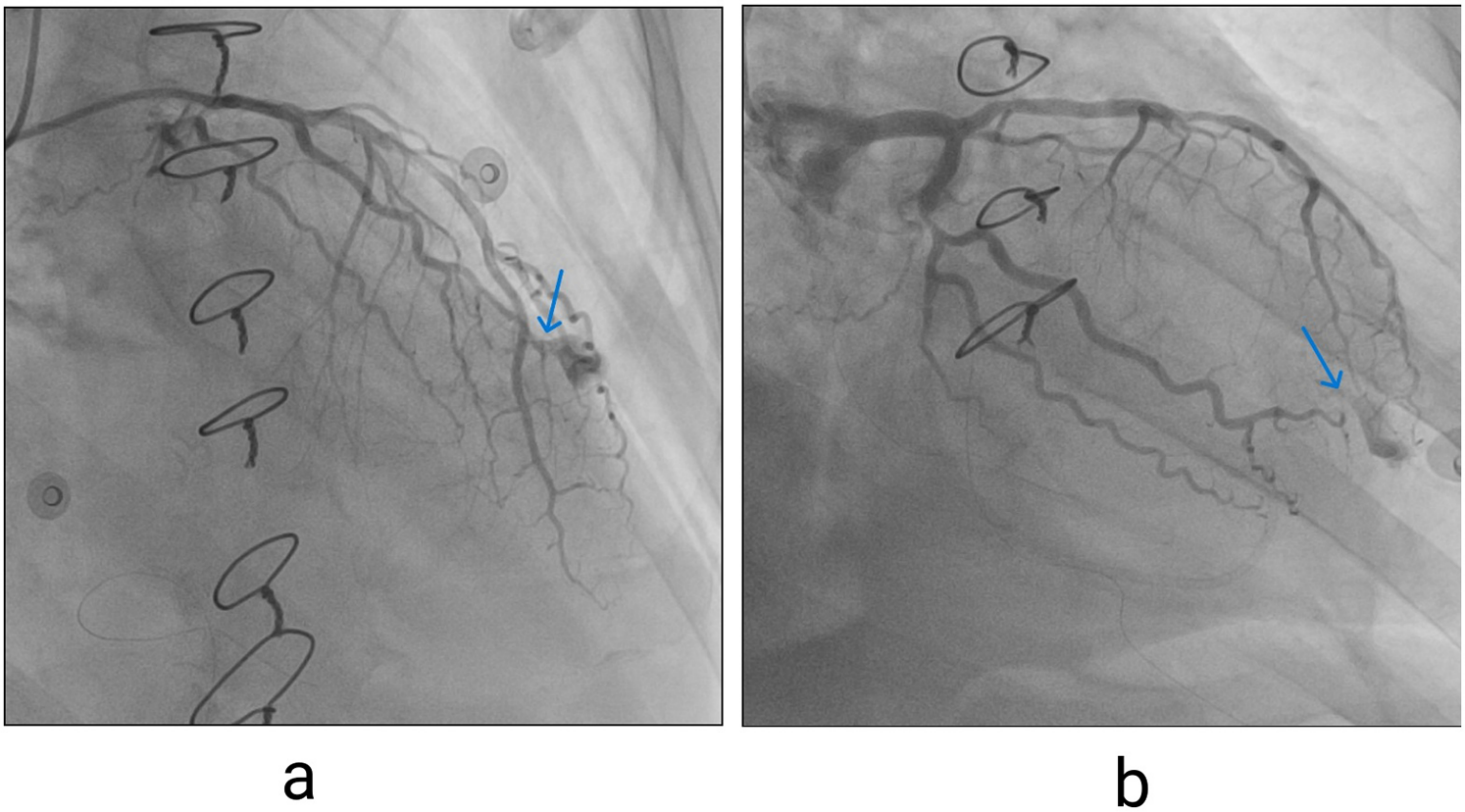
**(a)** Contrast extravasation seen on invasive coronary angiography, left coronary artery in right anterior oblique (RAO) cranial view, blue arrow pointing to extravasation site. **(b)** Left coronary artery in RAO caudal projections (blue arrow pointing to extravasation site).

The patient was rushed to the cath lab, where invasive coronary angiography was performed. Significant stenosis of the Cx was noted, and extravasation of contrast from the distal segment of OM1 was identified. (Figures 3a,b).

**Figure 4:**
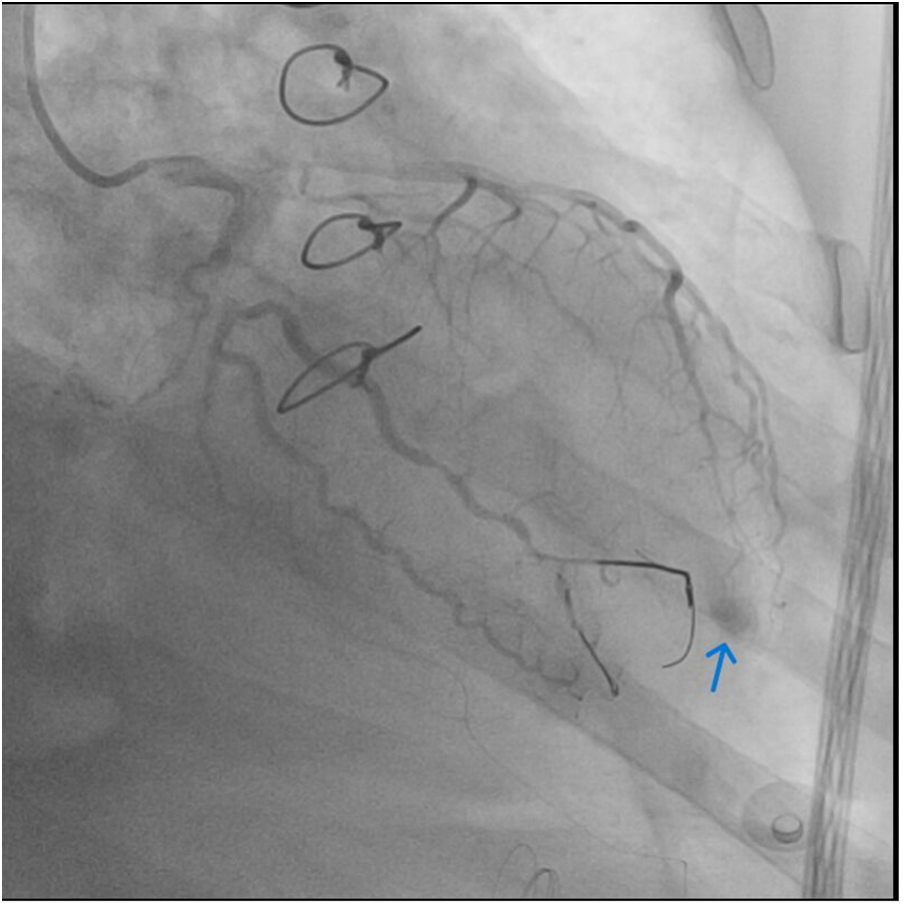
Coronary angiography—final result after placing guide wire tips in lacerated artery. No further contrast extravasation is seen. Small pool of contrast remains trapped in pericardium at the previous bleeding site.

Microcoil embolization was unsuccessful due to the lack of a device of adequate size. A different approach, using cut-off coronary guidewire tip segments advanced through a microcatheter, led to successful embolization of the OM branch and cessation of bleeding. (Figure 4).

After the procedure, the patient stabilized with no further signs of blood loss. A repeated echocardiogram in the following days showed regression of the pericardial effusion.

The patient was discharged home seven days post-embolization in stable condition.

## Follow-up and outcome

Five days after discharge, the patient was readmitted to our hospital due to worsening overall condition. Upon admission, he was somnolent, hypotensive, tachycardic, pale, oliguric and febrile.

Repeated echocardiography showed normal cardiac function and pericardial effusion, expanding alongside left ventricular wall with thickness from 22 mm–30 mm. There were no signs of increased intrapericardial pressure and heart chambers compromise. Since the pericardial effusion had higher echogenicity indicating highly viscous fluid similar to intrapericardial hematoma that probably could not be aspirated and there were no hemodynamic effects of the effusion, it was treated conservatively. Laboratory findings showed elevated levels of C-reactive protein and procalcitonin pointing out towards sepsis. After the administration of Cefepime, the condition stabilized over the next few days and he was discharged ten days later to a regional hospital.

## Discussion

Coronary artery perforation is a rare complication of pericardiocentesis, occurring in less than 1% of cases (2, 4). Unless, urgent pericardial drainage is required, CTA can provide valuable information. It can help to diagnose coronary artery injury and help determine the treatment strategy. In this case, CTA enabled us to diagnose distal OM injury and opt for a less invasive coronary embolization rather than a repeated thoracotomy.

Coronary CTA is recommended for patients with iatrogenic complications and hemopericardium if they are hemodynamically stable and can tolerate the examination. “Motion” artifacts can occur in patients with high heart rates, especially those in hemorrhagic shock, complicating CT image interpretation. Breath-holding and cessation of body movements are essential for high-quality imaging, which can be challenging in critically ill patients (5).

The early arterial phase and contrast extravasation in the apicolateral myocardium and epicardium raised suspicion of possible OM laceration.

The spatial resolution of the CT scan, made it difficult to distinguish small structures like the distal OM with a diameter of less than 1 mm. In such cases, using the portal venous phase to assess myocardial perfusion can be beneficial. Hypoperfusion of the anterolateral papillary muscle suggested OM perforation, resulting in reduced blood flow in its irrigation area. These findings were critical in shaping our treatment strategy.

Although coronary perforation as a complication of pericardiocentesis has been previously described it occurs more frequently in subxiphoid approach, while the lacerations occur more frequently in major epicardial coronary arteries like dominant RCA or dominant Cx. Iatrogenic injury to OM branch occurs rarely and can be overlooked due to its small diameter and contained perforation (6, 7). We had to use cut off coronary guidewire tips since conventional method of using micro coils was not available (8, 9).

## Conclusion

Rare complications after pericardiocentesis can be diagnosed using CT angiography and based on the findings, interventional strategy can be tailored to accommodate the patient's conditions striving for long term benefit. Interventional strategies need to be adjusted in order to achieve optimal result.

## Data Availability

The original contributions presented in the study are included in the article/supplementary material, further inquiries can be directed to the corresponding author.
