# Supplementary figures and images for: Computerized tomography angiography in diagnosing an obtuse marginal branch perforation after pericardiocentesis: a case report
Source: Front Cardiovasc Med. 2025 Mar 11;12:1535797. doi: 10.3389/fcvm.2025.1535797 (PMC11932988; doi:10.3389/fcvm.2025.1535797)

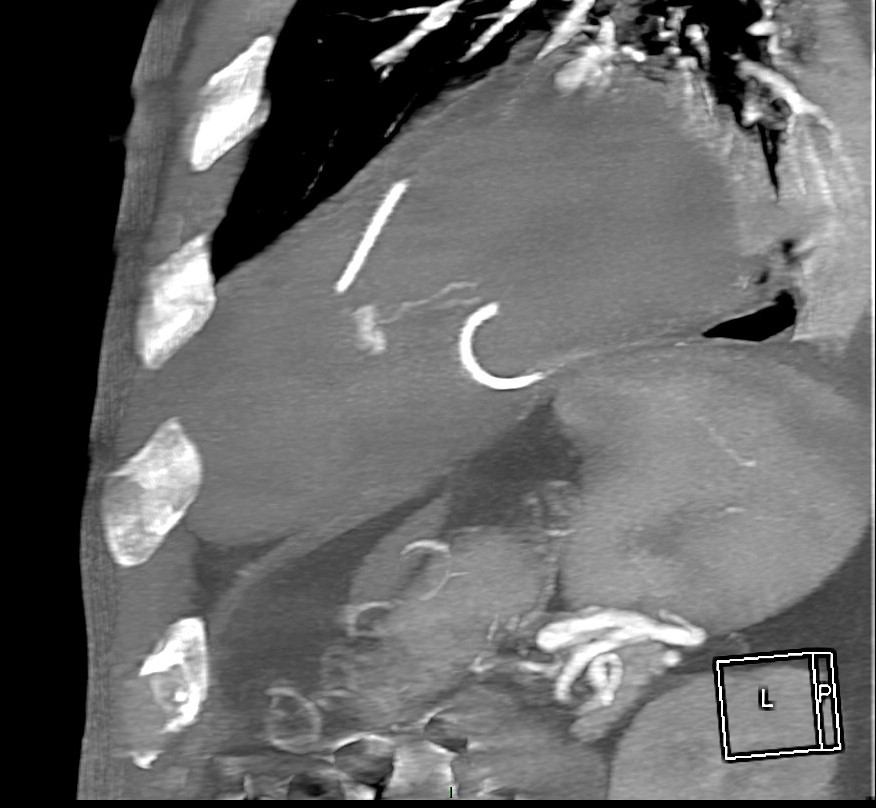

Supplement: Supplementary file 3 [file Image1.jpeg]

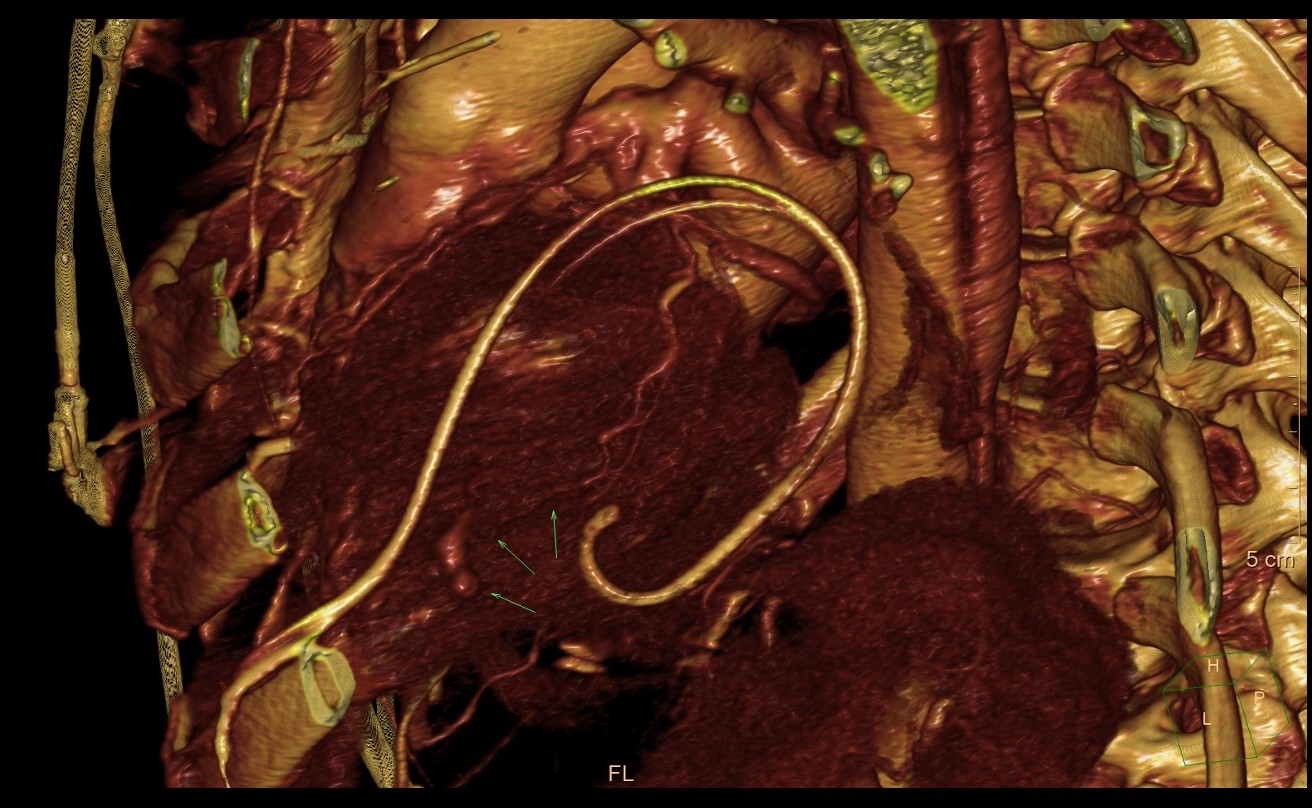

Supplement: Supplementary file 4 [file Image2.jpeg]
